# Drug-induced parkinsonism in a patient with DiGeorge syndrome: a case report

**DOI:** 10.3389/fnins.2024.1483587

**Published:** 2024-11-27

**Authors:** Clancy Cerejo, Nicolas De Cleene, Gerald Walser, Atbin Djamshidian, Klaus Seppi, Beatrice Heim

**Affiliations:** Department of Neurology, Medical University of Innsbruck, Innsbruck, Austria

**Keywords:** DiGeorge syndrome, parkinsonism, lamotrigine, early-onset, drug induced

## Abstract

DiGeorge syndrome, also referred as 22q11.2 deletion syndrome is a multisystem disorder associated with an increased risk of early-onset parkinsonism. In this case report, we present a case of a 47-year-old male patient with complex comorbidities and seizures. This patient presented with increased seizure frequency and on examination was found to have parkinsonism. Due to the symptoms constellation, a genetic analysis was done which revealed presence of DiGeorge syndrome. However, his DaTscan was normal and hence a possibility of medication induced parkinsonism was considered. Through this case report, we want to emphasize the fact that while it is important to consider genetic testing for young patients with parkinsonism especially in those with complex comorbidities, other possible causes of parkinsonism should not be ignored.

## Introduction

DiGeorge syndrome, also referred as 22q11.2 deletion syndrome (22q11.2DS), is a multisystem disorder caused by microdeletion of a 3 Mb segment on the long arm of chromosome 22. It is associated with increased risk of developing early-onset parkinsonism (Zaleski et al., 2009). This syndrome features with velocardiofacial abnormalities along with cognitive disabilities, psychotic disorders, immune deficiencies and parathyroid dysfunctions. Also, later-life complications can include schizophrenia, seizures and parkinsonism (Zaleski et al., 2009; Bassett et al., 2005). Here, we report a case of a middle-aged male with complex comorbidities, seizures and parkinsonian symptoms.

## Case report

**Figure 1:**
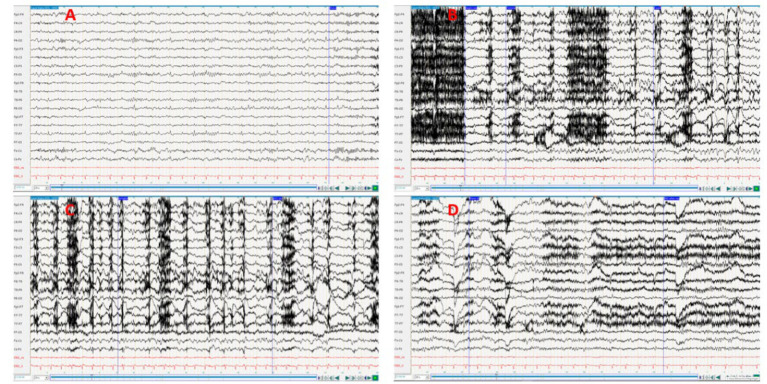
**(A)** Interctal; **(B,C)**, Ictal; **(D)** Postictal. The feed rate was set to 20 s to improve the visualization of the seizure. Medication during the EEG: Levetiracetam 1,500 mg 1-0-1 + Lamotrigin 100 mg 1-0—½. EEG interpretation: Pronounced muscle artifacts at the beginning of the episode, rhythmic theta activity with subsequent transition to delta activity on the right fronto-temporal, increasing in amplitude and spread over the right hemisphere and to the opposite side. The exact onset of ictal activity cannot be precisely determined due to the overlap with muscle artifacts (oroalimentary automatisms), duration <1 min.

A 47-year old right-handed Caucasian man with non-lesional right temporal lobe epilepsy (TLE) was admitted to our video-EEG-monitoring unit for subjective increase in seizure frequency. This patient first developed focal seizures at the age of 38. Diagnostic workup and tests back then (EEG, 3 T brain MRI, laboratory diagnostics, neuropsychological testing) were unremarkable and the diagnosis of non-lesional temporal lobe epilepsy was made (Figure 1). Due to intermittent breakthrough seizures, he has been treated with levetiracetam, lacosamide, and carbamazepine (Table 1). At the time of admission, he was on a combination of levetiracetam and lamotrigine.

**Table 1:** Antiseizure medication history.

Period	Antiseizure medications
Feb 2014	Levetiracetam (3 g), Lacosamide (150 mg)
June 2014–2020	Discontinued medications
June 2020	Carbamazepine (200 mg)
July 2020	Levetiracetam (2 g)
June 2022	Levetiracetam (3 g), Lamotrigine (450 mg)
October 2022–Present	Brivaracetam (200 mg), Lamotrigine (400 mg)

Furthermore, his past medical history included hypothyroidism, hypercholesterolemia, hypertension, hepatopathy (alimentary-toxic type), depressive symptoms, and valvular cardiomyopathy with mechanical aortic valve replacement at the age of 38. Family and birth history were unremarkable. He was born of non-consanguineous marriage. In the neuropsychological testing, he showed impaired performance in different executive functions with intact psychomotor processing speed. He studied up to secondary school and previously worked as a postman but has been registered as unable to work since the onset of recurrent seizures.

During the admission, the patient was alert and orientated. On neurological examination he had moderate hypomimia and hypophonia with reduced blink frequency and the lips were mildly parted when the mouth was at rest. Eye movements were normal. There was global slowness and paucity of spontaneous movements with bilateral rigidity and bradykinesia in both upper extremities with left emphasis. There were several interruptions on foot tapping bilaterally. Furthermore, there was intermittent distractible high-frequency and low-amplitude tremor of the eyelids and in all four limbs. Otherwise power, reflexes, sensation, pin-prick and cerebellar examination were normal and there were no pyramidal signs. He could get up from the chair instantly without assistance of the arms. He had a small stepped normal-based gait with bilateral reduced arm swing more pronounced on the left. The postural reflexes were intact.

**Figure 2:**
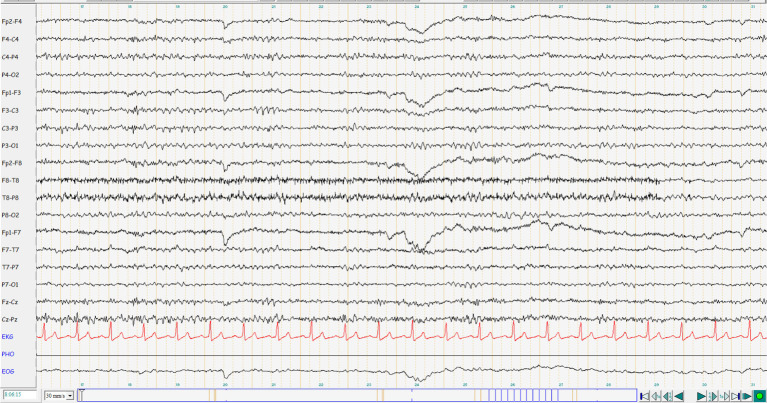
EEG interpretation: mildly abnormal EEG with diffuse theta enhancement. No potentials typical of epilepsy. Current medication: Brivaracetam 100 mg 1-0-1, Lamotrigine 200 mg 1-0-1.

During the 7 days of video-EEG recording, several habitual attacks with eyelid fluttering and subjective generalized weakness were recorded without epileptic correlate, which is why additional non-organic epileptic attacks were suspected. Also, due to a worsening of a pre-existing depression and the newly occurring psychogenic non-epileptic attacks while under treatment with levetiracetam, a switch to brivaracetam was made (Figure 2).

His routine blood evaluation revealed hypertriglyceridemia, raised liver enzymes, low haptoglobin, mildly raised hemoglobin and hematocrit value. He had a normal calcium, thyroid and iron function test. Further blood evaluation showed low IgM level, low absolute CD3 cell count and CD4/CD8 ratio.

**Figure 3:**
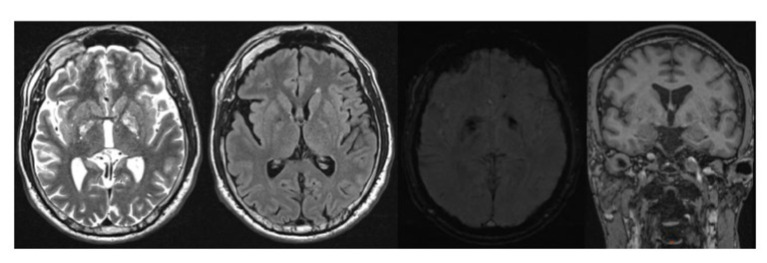
MRI Brain T2 weighted, FLAIR, SWI, T1 weighted images showing mild generalized cortical atrophy and iron deposits in the bilateral globus pallidus.

Because of the unclear TLE and newly diagnosed parkinsonism, we repeated the 3 T brain MRI, which showed increased iron deposits in the globus pallidus on both sides without further abnormalities (Figure 3). An expanded laboratory including a serum onconeural antibodies panel [anti-Yo, anti-Hu, anti-Ri, Anti-CV2, anti-Ma2, anti-amphiphysin, anti-TR(DNER), anti-SOX1, anti-Recoverin], neuronal surface protein antibodies panel [NMDA-R antibody, LGI1-antibody (VGKC), CASPR2-antibody (VGKC), AMPA-GluR1 antibody, AMPA-GluR2 antibody, GABAB-R antibody], 24-h urine copper and ceruloplasmin were normal. Ophthalmological examination showed no evidence of Kayser-Fleischer ring, optic atrophy or retinal degeneration, but a branch-vein occlusion of the left eye, for which intravitreal operative drug application was started. The patient was referred to an infectious disease specialist because of his low CD3-cell count, however in the absence of any current infection he was advised routine annual follow-up.

Due to the symptom constellation of non-lesional TLE, high-grade aortic valve insufficiency with cardiomyopathy and left-sided parkinsonism, a molecular genetic analysis using next generation exome sequencing method was performed which revealed 22q11.2 deletion syndrome with a heterozygous deletion of about 2.5 Mb on the long arm of chromosome 22. The phenotypic pattern of our 22q11.2DS patient mainly consisted of valvular cardiomyopathy, epilepsy, cognitive and psychiatric abnormalities, left-sided parkinsonism and hypothyroidism without prominent facial dysmorphism. Despite showing left-sided parkinsonism, the presynaptic dopaminergic transport scan was unremarkable. Theoretically, this could be due to possible non-dopaminergic-thalamocortical pathway dysfunctions triggering the parkinsonism (Błaszczyk, 2016). Another possibility could be drug induced parkinsonism. In our case, we could identify two potential drugs that could cause parkinsonian symptoms: levetiracetam and lamotrigine. However, a switch from levetiracetam to brivaracetam did not improve the parkinsonian symptoms. Hence, a possibility of lamotrigine induced parkinsonism was considered.

## Discussion

Parkinson’s disease (PD) is among the most prevalent neurodegenerative disorders worldwide. It manifests with motor symptoms like bradykinesia, rigidity and tremor, as well as non-motor symptoms like olfactory dysfunction, constipation, urinary incontinence, depression and cognitive impairments (Sveinbjornsdottir, 2016). The typical onset is later in life however occasionally in about 4% cases PD can occur at age < 50 years (Van Den Eeden, 2003). Of these early-onset PD cases, 22q11.2DS is now being identified as one such genetic cause leading to an increase risk of developing early onset PD (Boot et al., 2018).

Approximately 0.5% of early-onset PD are caused by 22q11.2DS (Boot et al., 2018). Several possible pathophysiological mechanisms are described that may play a role in 22q11.2DS-associated PD (Boot et al., 2018; Goldstein et al., 2014). One of the theories is the hyperdopaminergic mechanism leading to dopamine autotoxicity. The Catechol-O-methyltransferase (COMT) gene, involved in the degradation of catecholamines like dopamine, lies within the frequently deleted 3 Mb 22q11.2 deletion region. The 22q11.2 deletion patients are hemizygous for the COMT gene leading to a decreased level of the dopamine metabolite, indicating a hyperdopaminergic state. Another proposed mechanism is the mitochondrial dysfunction. In the commonly deleted 3-Mb 22q11.2 deletion region there are certain genes like PRODH, SLC25A1, MRPL40, TXNRD2, and TANGO2 that are involved in different aspects of mitochondrial function (Devaraju and Zakharenko, 2017). Reduced levels of these mitochondrial genes may also be a factor in the pathogenesis of parkinsonism in 22q11.2DS (Boot et al., 2018).

Based on the available literature most 22q11.2 DS PD patients, similar to our patient present with parkinsonian symptoms of bradykinesia, tremors and rigidity, but show early postural instability (Zaleski et al., 2009; Boot et al., 2018; Booij et al., 2010; Butcher et al., 2013; Dufournet et al., 2017). Often adults with 22q11.2DS are treated with neuroleptics, which hinder a reliable clinical diagnosis of PD. In such cases, DaT-scan can be useful to differentiate neuroleptic-induced parkinsonism from PD, and thus can also help to establish a possible etiologic association between 22q11.2DS and early-onset PD (Booij et al., 2010). Dufournet et al. (2017) has described five 22q11.2DS patients with early-onset PD whose DaT-scan showed presynaptic striatal denervation. However, the DaT-scan of our patient showed unremarkable presynaptic dopaminergic transport. Hence, a possibility of drug induced parkinsonism was considered.

As compared to the idiopathic Parkinson’s disease, drug induced parkinsonism usually presents with more symmetrical and bilateral symptoms with prominent bradykinesia and rigidity (Shin and Chung, 2012; Thanvi and Treadwell, 2009). Furthermore, in addition to rest tremor, patients often experience postural tremor (Shin and Chung, 2012). In our case, the potential drugs that could cause parkinsonism were levetiracetam and lamotrigine. Levetiracetam is a commonly used antiepileptic with an overall good safety profile. However, in recent literature, there are cases reporting levetiracetam as a rare cause of drug induced parkinsonism (Mathew et al., 2024). The exact mechanism of levetiracetam induced parkinsonism is not yet completely understood. Levetiracetam acts on synaptic vesicle protein SV2A and the L-type calcium channels in dopaminergic neurons which may interfere with dopamine release (Zesiewicz et al., 2005; Yan et al., 2013). Also the levetiracetam induced oxidative stress on cells can contribute in the neurodegenerative process observed in the dopaminergic system (Sarangi et al., 2016). In our patient, a switch from levetiracetam to brivaracetam failed to show improvement in the parkinsonian symptoms. Hence, a possibility of lamotrigine induced parkinsonism was considered.

Lamotrigine is reported to cause various types of movement disorders like parkinsonism, dystonia, dyskinesia, tremors, myoclonus, and tics (Rissardo and Fornari Caprara, 2021; Zipp et al., 1995; Shinotoh et al., 1997; Santens et al., 2006; Fleurat and Smollin, 2012). Until now only limited cases of lamotrigine induced parkinsonism are reported in the literature. Zipp et al. (1995) and Shinotoh et al. (1997), while evaluating the antiglutamatergic effect of lamotrigine for the symptomatic treatment of PD, were among the first to report lamotrigine induced worsening of parkinsonian symptoms in some of their study subjects. Santens et al. (2006) and Fleurat and Smollin (2012) each reported a case with seizure disorder on lamotrigine therapy who subsequently developed parkinsonian symptoms. In the case reported by Santens et al., lamotrigine was completed discontinued which resulted into near complete resolution of the parkinsonian symptoms, which further affirmed the correlation. Pathophysiologically it appears that lamotrigine stabilizes the presynaptic cell membranes thereby modulating the release of glutamate, aspartate, and GABA, which possibly modulates the brain dopamine levels (Rissardo and Fornari Caprara, 2021; Braga et al., 2002; Ahmad et al., 2004). A recent study found an association between lamotrigine, levetiracetam, and sodium valproate and incident parkinsonism, possibly due to the potential of these drugs to interfere in dopaminergic pathways (Belete et al., 2023). This is supported by a postmortem study showing reduced striatal levels of homovanillic acid and dopamine in patients with drug-induced parkinsonism (Rajput et al., 1982). However, the exact pathophysiological mechanism involved in lamotrigine induced parkinsonism still remains unclear.

In conclusion, our case reports a patient with 22q11.2 deletion syndrome with drug-induced parkinsonism. Genetic testing for 22q11.2 deletion syndrome should indeed be considered in early-onset Parkinson disease patients with complex comorbidities. Nevertheless, other possible causes of parkinsonism should not be ignored.

## Data Availability

The original contributions presented in the study are included in the article/supplementary material, further inquiries can be directed to the corresponding author.
